# Relationships in anesthesia work areas between contamination measured as colony-forming units, *Staphylococcus aureus* detection*, and S. aureus* transmission

**DOI:** 10.1007/s44254-026-00169-y

**Published:** 2026-04-07

**Authors:** Arnav Bhushan, Franklin Dexter, Carmen Brindeiro, Randy W. Loftus, Unyime Ituk

**Affiliations:** 1 Department of Anesthesia, University of Iowa, 200 Hawkins Dr, Iowa City, IA 52242 USA; 2 RDB Bioinformatics, Coralville, IA USA; 3 Mayo Clinic, Rochester, MN USA

**Keywords:** Anesthesia department, Hospital, Bacterial load, Equipment contamination, Infection control, Operating rooms, *Staphylococcus aureus*

## Abstract

**Purpose:**

Earlier, patients developed surgical site infection for 2.0% of cases without *Staphylococcus aureus* transmission through anesthesia work areas, 11% with *S. aureus* transmitted susceptible to prophylactic antibiotic, and 18% with transmission of antibiotic-resistant isolates. A randomized trial and an effectiveness study both found that anesthesiologists who used basic preventive measures (e.g., alcohol releasing intravenous caps) and received feedback on colony-forming units per surface area sampled (CFU) had reduced *S. aureus transmission* and postoperative healthcare-associated infections. We used prospectively collected data to evaluate whether CFU would be a reliable criterion for hospitals to assess anesthesiologists’ contributions to postoperative infections.

**Methods:**

During the summer of 2025, reservoirs (e.g., anesthetist’s hands at case start/end) were sampled during 81 cesarean delivery cases performed in the same operating room. There were ≤ 15 reservoirs sampled per case.

**Results:**

52/1016 reservoir samples had *S. aureus* detected, more often with greater CFU (*P* = 0.0063). The 159/1016 samples with < 100 CFU had no *S. aureus*. Total CFU of all reservoirs for each case to total *S. aureus* isolates was 2.50 × 10^9^ per *S. aureus* (standard error 0.53 × 10^9^, *N* = 81 cases). CFU and *S. aureus* transmission were uncorrelated (all 15 reservoirs’ unadjusted *P* ≥ 0.12, Holm-Bonferroni *P* > 0.99).

**Conclusions:**

With substantive contamination (≥ 100 CFU), so few isolates are *S. aureus* that surrogate measures of insufficient disinfection (e.g., ATP bioluminescence) are inaccurate markers both of *S. aureus* isolation and transmission. The lack of association between contamination and transmission shows that feedback on CFU provides information on the effectiveness of disinfection, not on *S. aureus* transmission.

**Supplementary Information:**

The online version contains supplementary material available at 10.1007/s44254-026-00169-y.

## Introduction

Recent large anesthesia clinical trials among patients undergoing major surgery reported surgical site infection rates of 8.6% [1], 6.7% [2], and 6.7% [3], respectively. Postoperative healthcare-associated infections occurred in 9.8% [4]. Most (64%) hospital patients receiving a parenteral antibiotic undergo an anesthetic [5].

Consider the "anesthesia work area" to include the patient's nose and skin, anesthesiologists' and anesthesia providers' hands, the anesthesia machine and cart, and intravenous lumens for administering anesthetic drugs [6]. The risk of development of surgical site infection was 2.0% (8/406) without *Staphylococcus aureus* transmission through the anesthesia work area, 11% (9/84) with transmission of *S. aureus* isolates that were susceptible to the prophylactic antibiotic used, and 18% (4/22) with transmission of prophylactic-antibiotic-resistant isolates (*P* < 0.001 for ordered association) [7]. *S. aureus* transmission in the anesthesia work area was associated with approximately 62% of surgical site infections, where 62% = (9 + 4)/(8 + 9 + 4) [7]. An example of *S. aureus* transmission was from the nasopharynx of one patient at the start of their case to hands of the anesthesiologist at the end of the case, to the nasopharynx of the next patient in the room that day, and to the anesthesia machine agent dial [8]. Six categories of infection prevention products collectively contributed to preventing anesthesiologists’ pathogen transmission: nasal antisepsis, skin antisepsis, hand sanitizers at the bedside, disinfecting IV caps, intraoperative decontamination, and feedback on colony-forming units per surface area sampled (CFU) [9, 10]. When anesthesiologists applied these measures, *S. aureus* transmission was reduced (relative risk 0.56, *P* < 0.001) and consequent surgical site infections by more than half (hazard ratio 0.12, *P* = 0.004) [11]. These anesthesiologist-led trial results were validated in an observational study by another anesthesia department across its entire surgical suite [12].

To apply these principles, the first step is to screen anesthetizing locations [13, 14]. For example, a hospital-based anesthesia group had 112 anesthetizing locations [14]. There was an average of approximately 21 new postoperative infections per week, diagnosed over 90 postoperative days by surgeons [15]. The 50% of anesthetizing locations with the fewest postoperative infections accounted for 5% of infections (99% confidence interval [CI] 1% to 10%) [14]. The 10% of locations with the most infections accounted for 40% of infections (99% CI 31% to 49%) [14]. Quality improvement and suitability for clinical trials starts with the hospital performing sampling at the start and end of cases in those operating rooms to determine for which, if any, anesthesiologists are contributing to those infections.

For the operating rooms with the most infections per week, samples are collected for pairs of consecutive cases on the same day in the operating room [6, 7, 10–12, 16]. The total collection times average 3.4 min before the start of surgery and 4.4 min at case end [6]. For the operating rooms with substantive *S. aureus* transmission, then greater attention is paid to patient nasal decolonization, anesthesia machine decontamination, anesthesiologists’ hand hygiene, etc., guided by CFU data [9, 11, 12]. For locations where the incidence of *S. aureus* transmission through the anesthesia work area is low (< 10%), the anesthesia team’s infection prevention role can be limited to administering antibiotics, preventing hypothermia, etc.

In the current study, we evaluated whether the initial screening for anesthesiologists’ roles in the pathogen transmission causing postoperative infections can be simplified to CFU alone and, if so, what CFU threshold to use. Hands, machine, stopcock, etc., are no less contaminated at the ends of cases than at their start, often the opposite [17–19]. Therefore, sampling could be limited to the end of the cases, halving the sampling effort. Furthermore, the clinical pathology would be vastly simpler: counting colonies, not speciating bacteria and matching them among reservoirs to evaluate whether they were transmitted.

The association between CFU, *S. aureus* isolation, and *S. aureus* transmission is unknown. On the one hand, CFU may have little association with *S. aureus* transmission. Measuring CFU is like using a paper log or phone application to assess whether a patient is taking a prescribed medication. The CFUs are assessing whether the process is being applied, not its efficacy; that is known from the earlier studies [11, 12]. On the other hand, in support of using CFU in lieu of *S. aureus* transmission when screening for anesthesiologists’ contribution to infections, we found earlier that ≥ 100 CFU in the anesthesia work area predicted *S. aureus* isolation [19]. When work area samples had ≥ 100 CFU, *S. aureus* were isolated from 6.3% (1339/21,105) [19]. In contrast, when < 100 CFU, *S. aureus* was detected in 0.8% (74/9500) [19].

The preceding retrospective cohort study used samples that underwent microbiological testing with many different thresholds for "too numerous to count [19]." Therefore, the relative population of all colonies (e.g., contributing to protein residue testing or adenosine triphosphate relative light value (ATP) [20]) versus *S. aureus* could not be estimated. Furthermore, the association between contamination and *S. aureus* transmission could not be determined because protocols and data varied among hospitals [10, 19]. In the current project, we used a homogeneous population of cesarean delivery patients at one hospital to quantify the relationships among contamination of the anesthesia work area, *S. aureus* detection, and *S. aureus* transmission.

## Methods

The University of Iowa Institutional Review Board determined on 4 December 2024 that this project, #202,411,594, does not meet the regulatory definition of human subjects research because it was a quality improvement project based on the assessment of cultures in a single operating room. The project was coordinated with the Obstetrics Quality & Safety Committee. Our microbiological study was feasible because the samples were collected during one procedure (81 cesarean deliveries) performed in a single operating room [21]. The project was performed during the summer of 2025 (May 27 through August 29), on 50 distinct regular workdays when research assistants were available [21]. Most cases (68) and days (43) were obtained by the medical student author (AB).

### Sampling reservoirs

OR PathTrac (RDB Bioinformatics, Coralville, IA) collection kits were used for microbiological sampling [11, 22]. In earlier studies, there have been *N* = 13 reservoirs sampled for each case [6, 7, 11, 12]. The adjustable pressure-limiting valve and agent dial of the anesthesia machine are sampled at case start and at case end (*N* = 2 samples). The hands of the attending anesthesiologist and the resident physician are sampled before the case begins and at the end of the case (*N* = 4 samples). Samples are obtained from the patient’s nares (both sides) at the start and end of the case (*N* = 2 samples), from the axilla (both sides) at the start and end of the case (*N* = 2 samples), and from the groin (both sides) after performing spinal anesthesia (before surgical skin antisepsis), and at case end before leaving the operating room (*N* = 2 samples). Three patient sites are used because of the resulting high (94%) sensitivity for detecting *S. aureus* [23]. The axilla is sampled because the anesthesia team can repeatedly be in contact during cases with arm extension, including during monitor placement. The groin is sampled because often colonized [23]. The surface of the closed, disinfectable stopcock is sampled at the end of the case (*N* = 1 sample). For the current study, there were *N* = 15 reservoirs sampled for each case. These were the 13 samples regularly obtained [6], plus the anesthesia electronic health record's computer mouse and the drug infusion pump digital surface, which were sampled at case start and end (*N* = 2 samples). The kits provided a step-by-step sampling workflow with pre-labelled packets for samples and barcodes. Only the barcodes and collection dates were recorded, not the patients, anesthesiologists, or anesthesia residents. Because no demographic or clinical information could be saved under this quality improvement protocol, specific reasons for uncollected samples (e.g., an awake patient declining a swab) were not recorded [21].

The adjustable pressure-limiting valve and agent dials of the anesthesia machine were included as one environmental sample [6, 11, 19]. A sterile swab was rolled several times over the entirety of these selected areas to obtain samples [6, 22], as these areas total < 100 cm^2^. Flocked ESwabs (Copan, Italy) were used without charging or water. Anesthesia machines and carts can be fully disinfected with wipes [24]. However, in practice, anesthesia providers at the University of Iowa have not achieved this level of disinfection on machines’ knobs, dials, and ridges [25]. During the project period, the wipes used contained hydrogen peroxide [26]. The same types of wipes were used to disinfect the anesthesia electronic health record mouse and the anesthesia medication cart drawer handles. We sampled those sites because elective cesarean deliveries were performed with spinal anesthesia, so we thought that the anesthesia machine sites might be poor markers for areas touched by contaminated hands. A single sterile swab was rolled over those surfaces for a total of approximately 100 cm^2^. For sampling the surfaces of the primary injection port used for medication administration, a smaller flocked swab was used to ensure that only surfaces in contact with syringe tips were sampled.

Hands of the anesthesiologist attending, and resident physician or nurse anaesthetist, were sampled by using the modified glove juice method [27, 28]. Specifically, all the contents of a 50 ml conical tube holding a balanced electrolyte solution were poured into a sterile sampling bag. The dominant hand of the person being sampled was placed into the bag. The liquid was massaged on and around the entire hand for one minute, up to the wrist. The participant removed their hand from the bag. The liquid was poured back into the tube. The tube was recapped. The participant dried their hand with a provided paper towel.

For sampling the patient, one sterile swab (Copan Diagnostic Inc., Corona, CA) was used to sample the anterior 1/3 of each nares by gently inserting the swab into the anterior 1/3 of the nares and fully rotating 10 times [22]. One swab was used to sample both axillae. The swab was inserted to the midpoint of the axilla and rotated 10 times. One swab was also used to sample both inguinal creases by rotating along the entire bikini line. That sampling was done at the start of the case, just before the nurses placed the Foley catheter.

### Microbiology

Practitioners' hand samples were spun for 15 min at 4,500 rpm, the supernatant discarded, the pellet resuspended in one mL of sterile deionized water, and vortexed on high for five seconds or until the suspension became turbid to ensure resuspension of the pellet [19]. A final serial dilution of 1:10,000 was prepared, and a one µL loop was introduced into the vortexed suspension and used to cover the entire surface of a sheep blood agar plate using a wagon-wheel technique [19]. Next, the sample was vortexed again at high speed for five seconds [19]. A ten µL loop was used to streak the first quadrant of mannitol salt, bile esculin azide, and MacConkey agar plates [19]. A ten µL loop was then used to streak the remaining surface of each plate using the four-quadrant technique [19]. All agar plates were then incubated aerobically at 36 ± 1 °C for 48 h [19].

All other samples were vortexed at high speed for five seconds or until turbid [19]. For the disinfectable stopcock reservoir samples, a ten µL loop was introduced into the vortexed liquid [19]. It was used to cover the entire surface of the sheep blood plate using a wagon wheel technique [19]. Environmental and patient reservoir samples were serially diluted to 1:1000, and a one µL loop was used for plating as described for the lumen samples. Next, each sample was vortexed again at high speed for five seconds [19]. The ESwab was pressed against the side of the tube to remove excess liquid [19]. The swab was used to cover the first quadrant of the mannitol salt, bile esculin azide, and MacConkey agar plates [19]. A ten µL loop was used to streak the remaining surface of each agar plate using the four-quadrant technique [19]. All agar plates were incubated aerobically at 36 ± 1 °C for 48 h [19].

CFU were enumerated from sheep blood agar plates after 24 h of incubation at 36 ± 1 °C. Counts were then adjusted to the appropriate dilution. Final dilutions were 10^–2^ for stopcock samples, 10^–6^ for all environmental and patient samples, and 10^–7^ for hand samples.

Colonies selected for further isolation were identified based on their characteristic growth on selective media. Yellow colonies on mannitol salt agar were considered presumptive *Staphylococcus* spp. All isolates that grew from MacConkey agar were also sub-cultured onto sheep blood agar plates and incubated for 24 h at 36 ± 1 °C [19].

Following incubation, isolates underwent a series of simple, rapid biochemical tests, including Gram-staining, fermentation, catalase, oxidase, indole, and slide coagulase assays [29]. *S. aureus* isolates were streaked to methicillin-inoculated plates to identify resistance.

*S. aureus* transmission was considered to have occurred during a case when there were two or more distinct, serially examined reservoirs from the same operating room on the same day that were both positive with the same antibiotic profile [7, 10–12, 22]. For *S. aureus* detection at case end in patient samples to be treated as a transmission event, all three [23] reservoirs (nose, groin, and axilla) had to be negative at case start. For patient reservoirs *S. aureus* transmission was also considered to have occurred for a case if there were one or more reservoirs from the same operating room on the same day that were negative at baseline while positive for *S. aureus* at case end.

### Statistical analyses

The sample size was based on our local quality improvement goal [21] of quantifying *S. aureus* transmission to learn whether it was sufficiently large (≥ 12% [12]) to contribute substantively to surgical site infections in the studied operating room [16]. To differentiate between a high incidence of *S. aureus* transmission (40.70%) and a low incidence (12.05%) [12, 16], the exact binomial sample size would be *N* = 15 pairs of consecutive cases [16]. To differentiate between high and moderate (20.48%) incidences, the sample size would be *N* = 35 case pairs (i.e., 70 cases). During the above-described summer period, we obtained samples from *N* = 81 cases and *N* = 31 case pairs [21].

First, we assessed the suitability of the data for the scientific studies by checking that results matched earlier findings from a study of 31,783 reservoir samples from 105 distinct operating rooms [19]. Reservoirs with greater CFU would be expected to have a greater probability of *S. aureus* detection. This hypothesis was tested, while addressing that reservoirs were nested within cases, by using a cluster-robust jackknife approach (leave-one-case-out) to compute the standard error and one-sided *P*-value for Spearman's rho (Stata v19.5, StatCorp, College Station, Texas). We used a one-sided *P*-value because, if there were no colonies, there was no *S. aureus* (i.e., there can either be a significant positive association or no association). The supplementary material has results for the exact Wilcoxon-Mann–Whitney test.

Second, the ratio of total CFU (per surface area sampled) to *S. aureus* isolates was estimated. The total of colonies and isolates among all reservoirs was analyzed for each case. The standard error for the ratio of the means was estimated using the jackknife estimator via the Stata ratio command. This resampling method was chosen to achieve a reliable estimate of the precision of the ratios without requiring the assumption that the large bacterial counts follow normal distributions.

Third, for each of the 15 measured reservoirs' CFU, we estimated the association with overall *S. aureus* transmission. By doing so, we evaluated whether CFU or related markers, such as ATP [20], could serve as reasonably accurate surrogates for *S. aureus* transmission in the anesthesia work area. The rationale for the hypothesis would be that if *S. aureus* is invariably present when there are enough bacteria, and *S. aureus* is invariably transmitted when there are enough of them, there should be an association. The countervailing argument would be that *S. aureus*, and especially hypertransmissible sequence types, are distinct organisms. Because the 15 reservoirs were obtained from the same cases, a Holm-Bonferroni adjustment was applied to the 15 comparisons, treating the one-sided exact Wilcoxon-Mann–Whitney-adjusted *P* < 0.05 as significant. Holm-Bonferroni adjustment is applied sequentially, controlling the probability of making at least one false positive conclusion while generally obtaining greater statistical power than a simple Bonferroni correction. We considered one-sided tests because CFU associated with less *S. aureus* transmission (e.g., from a microbiome type effect) would not result in the clinical intervention of deliberately contaminating anesthesia work areas. We analyzed each reservoir individually because CFUs are used to make recommendations for targeted improvements to each reservoir individually. For example, consider the measurement of the anesthesia machine at start of case 1. If regularly ≥ 100 CFU, terminal cleaning of the room the night before should be improved. The improvement target is unrelated to the other 14 sampled reservoirs. To quantify the aggregate value of CFU to predict *S. aureus* transmission in anesthesia work areas, we calculated the arithmetic mean of the 15 reservoir-specific estimated probabilities that greater CFU’s were associated with transmission, weighting each reservoir equally. We calculated the standard error and 95% CI for this pooled mean using the jackknife procedure with clustering by case.

## Results

**Fig. 1:**
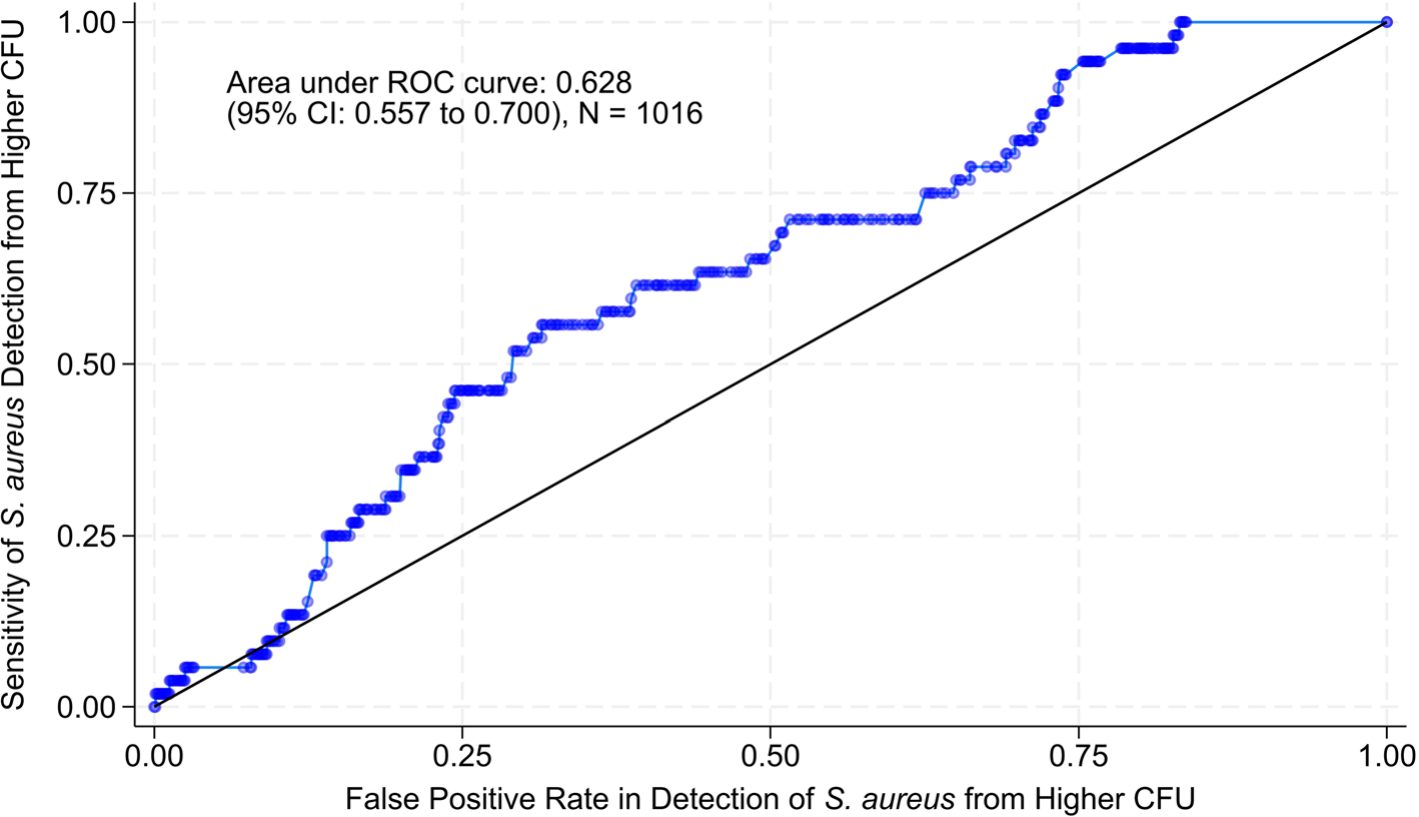
Receiver operating characteristic curve evaluating the ability of higher colony-forming unit (CFU) counts to predict *Staphylococcus aureus* detection. The plot includes the *N* = 1016 sampled reservoirs, illustrating the sensitivity and false-positive rates across varying CFU thresholds. *CI* confidence interval, *ROC* receiver operating characteristic

Across the 81 cases’ 1,215 possible reservoir samples (15 per case), *N* = 1016 were collected. There was a mean of 9.21 × 10^7^ CFU (standard deviation 1.28 × 10^8^) among the 310 environmental reservoir samples; 25th 50th, and 75th percentiles of 0.80, 5.10, and 12.30 × 10^7^ [30]. *S. aureus* isolates were detected in 9 of the 290 environmental samples with ≥ 100 CFU per surface area sampled. There was no (0) *S. aureus* detected among the 20 environmental samples with < 100 CFU. Among the environmental samples from which *S. aureus* was isolated, all had CFU ≥ 5000. Using all *N* = 1016 reservoirs' samples, there were 52 (5.1%) with *S. aureus* detected, more often among the reservoirs with greater CFU (*P* = 0.0063, area under the receiver operating characteristic curve 0.628 [95% CI 0.557 to 0.700]; Fig. 1). There was no *S. aureus* detected among the 159 reservoir samples with < 100 CFU. Among the reservoir samples from which *S. aureus* was isolated, all had CFU ≥ 500. These observations match earlier findings that ≥ 100 CFU in the environment, specifically the anesthesia machine, at the start and end of cases predicted *S. aureus* isolation [19], demonstrating the generalizability and validity of the current microbiological samples from a single operating room.

Unlike in earlier studies, successive dilutions were used so that there were not "too numerous to count" CFU [19]. The ratio of total CFU of all sampled reservoirs for each of the *N* = 81 cases to each case’s total (53) *S. aureus* isolates was estimated pairwise by case using the jackknife estimator. The anesthesia work area had an overall count of 2.50 × 10^9^ colonies per *S. aureus* (standard error 0.53 × 10^9^, 95% CI 1.44 × 10^9^ to 3.56 × 10^9^). For context on the estimated ratio, the combined population of Europe and Africa in 2023 versus one person was comparable: 2.29 × 10^9^ to one [31]. Therefore, although small CFU indicative of disinfection were associated with the absence of *S. aureus* [19], markers related to total bacterial populations, such as ATP or protein residue testing, would be inaccurate surrogates.

Because there cannot be *S. aureus* transmission without *S. aureus*, could rapid assays [32] for *S. aureus* detection be interchangeable with assessing transmission? Conceptually this would be so because, for the intravenous lumen at the end of the case, any *S. aureus* detected were transmitted given that intravenous lumens start sterile. Among the seven reservoirs sampled at the start of cases, *S. aureus* was detected in 18 of 81 cases. Of the 18 cases, 10 had no detected transmission (false positive 56%). Among the eight reservoirs sampled at the end of cases, *S. aureus* was detected in 23 of 81 cases. There were 6 of these 23 cases with isolates but without transmission (false positive 26%). Combined, 10 of 28 cases with *S. aureus* isolates lacked transmission (false-positive rate 36%). Thus, although *S. aureus* transmission through the anesthesia work area determined in this manner has been associated with postoperative healthcare-associated infection [7, 11, 12], and there cannot be *S. aureus* transmission without the presence of isolates, *S. aureus* detection is an inaccurate measure of transmission.

Combining results, we would expect no association between contamination of each of the 15 reservoirs (CFU per surface area swabbed) and *S. aureus* transmission in the case. The sample sizes ranged from *N* = 52 for the patient’s nose at the end of the case to *N* = 81 for the intravenous lumen at the end of the case, anesthesia machine at the start of the case, and anesthesia machine at the end of the case. All 15 unadjusted one-sided Wilcoxon-Mann–Whitney *P* ≥ 0.058, and all Holm-Bonferroni adjusted *P* > 0.99. This finding of an absence of association between greater contamination and greater risk of *S. aureus* transmission seems reliable, given that 11 of 15 reservoirs showed greater observed contamination among cases without transmission (e.g., patients' groins at case start; two-sided unadjusted *P* = 0.012, *N* = 53 cases). The mean (standard deviation) of the 15 estimated probabilities that greater CFU were associated with transmissions was 0.453 (0.090). Applying the jackknife to account for the hierarchical clustering of reservoirs within cases, the overall estimated mean effect size was 0.453 (standard error 0.036). The 95% CI of 0.38 to 0.52 includes the null value of 0.50 (*P* = 0.19), further suggesting the absence of a systematic association.

## Discussion

When anesthesia clinicians received feedback days to weeks later regarding CFU ≥ 100 for reservoirs in the anesthesia workspace, they subsequently identified improvement targets in basic infection control measures, reducing *S. aureus* transmission and surgical site infections postoperative healthcare associated infections [6–19]. The current study showed such a weak association between contamination and *S. aureus* transmission that, functionally, no association can be expected for individual operating rooms. When there is substantial contamination (≥ 100 CFU) [19], *S. aureus* is such a tiny fraction of bacteria in the anesthesia work area that surrogate measures of insufficient disinfection (e.g., ATP bioluminescence [20]) are likely inaccurate markers not only of *S. aureus* transmission but also of *S. aureus* isolates. These results are especially useful because quantified ATP were themselves correlated with CFU (*P* < 0.001), but the association itself was weak (e.g., Spearman rank correlation 0.244, *N* = 400) [20]. These results also help us understand how anesthesia clinicians reduced surgical site infections in earlier studies [11, 12]. In clinical trials and regular use, "feedback" to anesthesia clinicians referred to information on CFU by reservoir, because every reservoir sample has an associated CFU [11, 12]. Feedback on *S. aureus* transmission for each operating room can be provided too, and has been, but requires weeks of data because, hopefully, transmission is at a desired low incidence (e.g., 12% [16]) and operating rooms are the unit of monitoring, not specialties, because operating rooms differ in the specific anesthesia machine, cart, etc. [33]. (Feedback on postoperative healthcare-associated infections takes months by room.) Therefore, daily information on CFU's provides information on the effectiveness of the interventions helping anesthesiologists prevent surgical site infections (e.g., the anesthesia equipment is being wiped appropriately [24, 25] and effectively [26], chlorhexidine is actually being applied to the patient's groin and axilla [34], and disinfecting caps are actually being used [35, 36]).

Rapid testing for *S. aureus* has been developed, although optimization is pending [32]. Our results suggest the role that rapid testing can eventually play for monitoring *S. aureus* through anesthesia work areas. CFU monitoring for feedback to anesthesiologists about practices in specific operating rooms has no association with the care of individual patients [11, 12]. Rather, ≥ 100 CFU shows improvement targets for future reductions in contamination and *S. aureus* transmission. *S. aureus* transmission monitoring as used in the current study also does not have relevance to the care of individual patients because what is determined is whether there has been any *S. aureus* transmission through the anesthesia work area for the case, usually not the pathway. In the future, rapid testing for *S. aureus* might change the strategy. Patients who develop postoperative healthcare-associated infections are mostly those who stayed at least overnight in hospitals [37]. Results known within 1 or 2 h could be used to guide interventions such as reapplying (or applying, as for cesarean delivery patients) nasal povidone-iodine, chlorhexidine, or changing or removing contaminated intravenous catheters. Before the current study, it was not known whether *S. aureus* detection might be sufficient information compared with rapid testing of all reservoirs to assess transmission. Results from the current study show that many of the isolates detected are not transmitted (i.e., transmission should be determined). Earlier observations made this result expected, but, as far as we know, it has not been previously quantified [38–40]. Specifically, some *S. aureus* sequence types, including ST 5, exhibit biofilm and desiccation tolerance, contribute to a greater probability of transmission (estimated incidence risk ratio 6.7, *P* = 0.0008) [38, 39]. Likewise, clonal transmission of *S. aureus* including ST 5 was associated with the probability of transmission (risk ratio 3.2, *P* = 0.0006) [40]. Those comparisons of transmitted versus not transmitted *S. aureus* isolates suggest, in retrospect, the generalizability of the current study’s findings that multiple *S. aureus* are not transmitted.

Our study was limited in that the validity of using temporal association for assessing intraoperative *S. aureus* was not replicated but based on thousands of patients in multiple earlier studies [7, 9–12, 38–40]. Concurrent validity was shown based on the precedingly described verification using the gold standard of molecular typing of individual bacteria [38–40]. Predictive validity was shown, as described in the Introduction, based on the detected *S. aureus* transmission predicting the future outcome of surgical site infection [7]: 2.0% (8/406) without transmission, 11% (9/84) with detection of *S. aureus* transmission of isolates susceptible to the prophylactic antibiotic used, and 18% (4/22) with transmission of resistant isolates (*P* < 0.001 for ordered association). Construct validity was established based on interventions (e.g., anesthesiologist hand hygiene) reducing CFU resulting in reduced measured *S. aureus* transmission, and infection [9–12].

Our study was limited to *S. aureus* in anesthesia work areas. The operating room studied was, in retrospect, especially suitable because it had considerably more *S. aureus* detected in reservoir samples (5.2%) than in multiple earlier studies (0.7% [220/31783]) [19]. Regarding *Enterococcus*, not only is *S. aureus* transmission through anesthesia work areas more common than *Enterococcus* transmission (odds ratio 3.5, *P* < 0.0001), but *Enterococcus* lacks a direct route of transmission into the patient because transmission rarely involves the intravenous lumen (odds ratio 33.5, *P* < 0.0001) [10]. Furthermore, among 389 *Enterococcal* isolates from anesthesia work areas, 54% (210/389) were detected on future dates [41]. The vancomycin-resistant *Enterococcus* isolates were less often associated with spread within the same room daily than sensitive isolates (*P* = 0.002) [41]. The transmitted isolates were not associated with postoperative healthcare-associated infections (*P* = 0.23) [41]. Regarding gram negatives (e.g., *Pseudomonas aeruginosa*) in the anesthesia environment, *S. aureus* has transmission pathways that are vastly more common than those of the gram-negative pathogens (odds ratio 8.5, *P* < 0.0001) [10]. Unlike *S. aureus*, few (6% [84/1448]) gram-negative isolates are detected on subsequent dates, and of those, 0% were piperacillin-tazobactam-resistant [41]. None (0%) of those (few) transmission events in the anesthesia work area were associated with postoperative healthcare-associated infection [41].

In conclusion, ratios of all bacteria to *S. aureus* in anesthesia work area samples were more than a billion to one. Therefore, when there is substantive contamination (≥ 100 CFU) [19], *S. aureus* is such a tiny fraction of bacteria that surrogate measures of insufficient disinfection (e.g., ATP bioluminescence [20]) would be inaccurate markers of *S. aureus* counts, nonetheless transmission. There were no associations between contamination of each of the 15 reservoirs (CFU per surface area swabbed) and *S. aureus* transmission (all adjusted *P* > 0.99). Therefore, in clinical trials and regular use, "feedback" to anesthesia clinicians refers to information on CFU and the effectiveness of the disinfection approaches for each reservoir [11, 12]. Such feedback should not be misinterpreted as being about *S. aureus* transmission. Results suggest that hospitals should not wait weeks for sufficient transmission events; rather, they should promptly provide the daily CFU data. Second, when screening operating rooms with high rates of postoperative healthcare-associated infections [13, 15] to assess the contribution of anesthesia clinicians to those infections, *S. aureus* transmission needs to be measured, which is the current protocol [16]. Neither CFU nor even *S. aureus* detection were sufficient surrogates.

## Supplementary Information

Supplementary Material 1.

## Data Availability

Stata computer code and output in sequence of the Results are included in the supplementary material. The corresponding data files are available after data use agreement with the University of Iowa (click here). Contact Dr. Dexter who will coordinate. The variables are defined in the Stata describe statements in the output on lines 4 and 57.

## Abbreviations

ATP: Adenosine triphosphate
CFU: Colony-forming units
CI: Confidence interval
