# Supplementary material for: Relationships in anesthesia work areas between contamination measured as colony-forming units, Staphylococcus aureus detection, and S. aureus transmission
Source: Anesthesiol Perioper Sci. 2026 Apr 7;4(1):22. doi: 10.1007/s44254-026-00169-y (PMC13056790; doi:10.1007/s44254-026-00169-y)
Supplement: Supplementary file 1 — Supplementary Material 1. [file 44254_2026_169_MOESM1_ESM.pdf]

```

version 19.5
describe, fullnames

* First paragraph of the Results
quietly summarize Env_CFU, detail   // median [quartiles] added per Reviewer #4 comment #4 R2
display "mean " %9.2e r(mean) " SD " %9.2e r(sd) " 25th " %9.2e r(p25) " median " %9.2e r(p50) " 75th " %10.3e r(p75)

tabulate  bEnv_CFU_100 bENV_SA       // SA: Staphylococcus aureus
summarize Env_CFU if   bENV_SA == 1

tabulate  All_SA

spearman  All_SA All_CFU, exact(montecarlo, reps(50000) rseed(123) nodots)
    display r(p_exact)/2 // one-sided
* Reviewer #2 comment #3 requests clustering by case
codebook BoxNumber, compact         // confirming this is unique identifier
jackknife rho=r(rho), cluster(BoxNumber): spearman All_SA All_CFU
display %5.4f r(table)[4,1]/2

replace             All_SA = 1 if All_SA > 1
ranksum All_CFU, by(All_SA) porder // confirming same so that can interpret with ROC AUC
display %4.3f 1-r(porder)
capture noisily ranksum All_CFU, by(All_SA) exact // shows why using spearman command

tabulate  bAll_CFU_100 All_SA
summarize  All_CFU  if All_SA == 1

roctab All_SA All_CFU, summary // added as recommended by Reviewer #2 comment #5
display "r(ub) - invnormal*r(se) = " r(ub) - ( invnormal(1-0.05/2)*r(se) + r(area) ) // asymptotic SE not extra info

* Editor requested figure in R2 review
roctab All_SA All_CFU, graph ///
    ytitle("Sensitivity of {it:S. aureus} Detection from Higher CFU")                  ///
    xtitle("False Positive Rate in Detection of {it:S. aureus} from Higher CFU")       ///
    note("") msize(small) mcolor("blue%30") mlcolor("blue")                            ///
    text(0.95 0.05 "Area under ROC curve: 0.628" "(95% CI: 0.557 to 0.700), N = 1016", ///
                   placement(se) justification(left) margin(small))
graph export "Figure 1 v20.jpg", as(jpg) name("Graph") replace width(3600) height(2160) quality(100)



* Second paragraph of the Results
summarize                     Total_Saureus
display "Total among all samples: " r(mean)*r(N)

ratio (Total_CFU/Total_Saureus), vce(jackknife, nodots)
matrix list r(table) // extra digits and confidence interval
```

```
display %4.2f r(table)[1,1] / 1E9
display %4.3f r(table)[2,1] / 1E9

* Reviewer #2 comment #3 inquires of inhierarchical structure. Adding to show effect (not in the results)
ratio (Total_CFU/Total_Saureus)
display %4.2f r(table)[1,1] / 1E9
display %4.3f r(table)[2,1] / 1E9



* Third paragraph of the Results
use "Temp_transmission.dta", clear
describe, fullnames

generate b_any_transm = (total_transm > 0) if total_transm != .
generate b_Sa_Start   = (Sa_Start > 0) if Sa_Start != .
generate b_Sa_End     = (Sa_End   > 0) if Sa_End   != .

tabulate b_any_transm b_Sa_Start, row
tabulate b_any_transm b_Sa_End, column

generate b_Sa_Either = 0
replace  b_Sa_Either = . if Sa_Start == . & Sa_End == .
replace  b_Sa_Either = 1 if Sa_Start >= 1 | Sa_End >= 1
tabulate b_any_transm b_Sa_Either, column



* Fourth paragraph of the Results
* One-sided tests because would not deliberately contaminate to prevent S. aureus transmission
generate byte ms_msra_transm_any = 0
replace       ms_msra_transm_any = 1 if mssa_transm == 1 | mrsa_transm == 1

ranksum postmouseomnicell_cfu, by(ms_msra_transm_any) porder
display "unadjusted "             r(p_exact)/2
display "adjusted " min(0.999, 15*r(p_exact)/2)
ranksum ahoe_cfu, by(ms_msra_transm_any) porder
display "adjusted " min(0.999, 14*r(p_exact)/2) // Holm-Bonferroni sequential
ranksum aao1_cfu, by(ms_msra_transm_any) porder
display "adjusted " min(0.999, 13*r(p_exact)/2)
ranksum aho1_cfu, by(ms_msra_transm_any) porder
display "adjusted " min(0.999, 12*r(p_exact)/2)
ranksum pge_cfu , by(ms_msra_transm_any) porder
display 1 - (r(p_exact) / 2)                    // one-sided and reversing
ranksum premouseomnicell_cfu , by(ms_msra_transm_any) porder
display 1 - (r(p_exact) / 2)
ranksum pa1_cfu , by(ms_msra_transm_any) porder
display 1 - (r(p_exact) / 2)
```

```
ranksum pne_cfu , by(ms_msra_transm_any) porder
display 1 - (r(p_exact) / 2)
ranksum aaoe_cfu, by(ms_msra_transm_any) porder
display 1 - (r(p_exact) / 2)
ranksum pn1_cfu , by(ms_msra_transm_any) porder
display 1 - (r(p_exact) / 2)
ranksum le_cfu  , by(ms_msra_transm_any) porder
display 1 - (r(p_exact) / 2)
ranksum vdo1_cfu, by(ms_msra_transm_any) porder
display 1 - (r(p_exact) / 2)
ranksum vdoe_cfu, by(ms_msra_transm_any) porder
display 1 - (r(p_exact) / 2)
ranksum pae_cfu , by(ms_msra_transm_any) porder
display 1 - (r(p_exact) / 2)
ranksum pg1_cfu , by(ms_msra_transm_any) porder
display "two-sided " r(p_exact)
display 1 - (r(p_exact) / 2)


* Added for Reviewer #2 comment #3 requesting effect sizes in results
capture program drop mean_sd_auc
quietly {
    program mean_sd_auc, eclass // from Gemini 3 Pro, 13 Feb 2026

        syntax [if] [in]        // required for subsequent "jackknife" call
        marksample using

        quietly count if `using'
        local n_records = r(N)

        tempname sum_x sum_sq_x n_vars
        scalar `sum_x'    = 0
        scalar `sum_sq_x' = 0
        scalar `n_vars'   = 0

        foreach var of global cfu_vars {
            capture quietly ranksum `var' if `using', by(ms_msra_transm_any) porder // to get AUC
            if _rc == 0 {                         // needed here because N=52 to N=81 among reservoirs
                scalar `sum_x'    = `sum_x'    +  r(porder)
                scalar `sum_sq_x' = `sum_sq_x' + (r(porder)^2)
                scalar `n_vars'   = `n_vars'   + 1
            }
        }

        tempname         b
        matrix          `b' = (1 - `sum_x' / `n_vars') // for probability greater CFU with transmission
        matrix colnames `b' = mean_auc
        ereturn post    `b', obs(`n_records') esample(`using')
```

```
        ereturn scalar sd_auc = sqrt( (`sum_sq_x' - (`sum_x'^2)/`n_vars') / (`n_vars' - 1) )
    end
}

ds *_cfu                        // accumulates the 15 CFU variables (i.e., reservoirs)
global cfu_vars `r(varlist)'  // what used in the preceding program
mean_sd_auc                     // running the program once for (new) second to last sentence
display "Mean of the 15 AUCs: " %6.3f _b[mean_auc] " SD: "%6.3f e(sd_auc) " N cases as check: " e(N)
jackknife _b[mean_auc] : mean_sd_auc // new final sentence of the last paragraph of the results
test _jk_1 = 0.5


```

```
1 .

2 .
3 . version 19.5

4 . describe, fullnames

  Contains data
   Observations:         1,016
      Variables:             9
  -----------------------------------------------------------------------------------------------------
  Variable      Storage   Display    Value
      name         type    format    label      Variable label
  -----------------------------------------------------------------------------------------------------
  Total_CFU       double  %10.0g                Total CFU by Box Number (patient), N = 81 records
  Total_Saureus   byte    %10.0g                Total S aureus by Box Number
  Env_CFU         long    %10.0g                Environmental reservoir CFU, N = 310
  bEnv_CFU_100    byte    %10.0g                1 = Env CFU >= 100; 0 otherwise
  bENV_SA         byte    %10.0g                1 = Env reservoir with S. aureus; 0 otherwise
  All_CFU         long    %10.0g                CFU all reservoir samples, N = 1016
  bAll_CFU_100    byte    %10.0g                1 = All_CFU >=100; 0 otherwise
  All_SA          byte    %10.0g                S. aurues all reservoir samples
  BoxNumber       str6    %9s                   Box Number of each reservoir sample, N = 1016
  -----------------------------------------------------------------------------------------------------
  Sorted by:
       Note: Dataset has changed since last saved.
```

```
 5 .
 6 . * First paragraph of the Results
 7 . quietly summarize Env_CFU, detail   // median [quartiles] added per Reviewer #4 comment #4 R2

 8 . display "mean " %9.2e r(mean) " SD " %9.2e r(sd) " 25th " %9.2e r(p25) " median " %9.2e r(p50) " 75th " %10.3e r(p75)
```
mean 9.21e+07 SD 1.28e+08 25th 8.00e+06 median 5.10e+07 75th 1.230e+08

```
 9 .
10 . tabulate  bEnv_CFU_100 bENV_SA       // SA: Staphylococcus aureus
```

1 = Env CFU >= 100; 0 otherwise	1 = Env reservoir with S. aureus; 0 otherwise		
	0	1	Total
0	**20**	**0**	**20**
1	**281**	**9**	**290**
Total	**301**	**9**	**310**

```
11 . summarize Env_CFU if   bENV_SA == 1
```

Variable	Obs	Mean	Std. dev.	Min	Max
Env_CFU	**9**	**1.63e+08**	**1.38e+08**	**5000**	**3.42e+08**

```
12 .
13 . tabulate  All_SA
```

S. aurues all reservoir samples	Freq.	Percent	Cum.
0	**964**	**94.88**	**94.88**
1	**51**	**5.02**	**99.90**
2	**1**	**0.10**	**100.00**
Total	**1,016**	**100.00**	

```
14 .
15 . spearman  All_SA All_CFU, exact(montecarlo, reps(50000) rseed(123) nodots)

   Number of observations =  1,016
           Spearman's rho = 0.0979

   Test of H0: All_SA and All_CFU are independent
                     Prob = 0.0017
               Exact prob = 0.0017 (50,000 Monte Carlo permutations)

16 .     display r(p_exact)/2 // one-sided
   .00086

17 . * Reviewer #2 comment #3 requests clustering by case
18 . codebook BoxNumber, compact         // confirming this is unique identifier

   Variable    Obs Unique  Mean  Min  Max  Label
   ------------------------------------------------------------------------------------------------------------------------------
   BoxNumber  1016     81     .    .    .  Box Number of each reservoir sample, N = 1016
   ------------------------------------------------------------------------------------------------------------------------------

19 . jackknife rho=r(rho), cluster(BoxNumber): spearman All_SA All_CFU
   (running spearman on estimation sample)

   Jackknife replications (81): .........10.........20.........30.........40.........50.........60.........70.........80. done

   Jackknife results                                        Number of obs = 1,016
                                                            Replications  =    81

         Command: spearman All_SA All_CFU
             rho: r(rho)
             n(): r(N)

                                  (Replications based on 81 clusters in BoxNumber)
   ------------------------------------------------------------------------------
                |              Jackknife
                | Coefficient  std. err.      t    P>|t|     [95% conf. interval]
   -------------+----------------------------------------------------------------
            rho |   .0979116   .0383748     2.55   0.013     .0215434    .1742798
   ------------------------------------------------------------------------------
```

```
20 . display %5.4f r(table)[4,1]/2
   0.0063

21 .
22 . replace             All_SA = 1 if All_SA > 1
   (1 real change made)

23 . ranksum All_CFU, by(All_SA) porder // confirming same so that can interpret with ROC AUC

   Two-sample Wilcoxon rank-sum (Mann–Whitney) test

         All_SA |      Obs    Rank sum    Expected
   -------------+---------------------------------
              0 |      964    483759.5      490194
              1 |       52     32876.5       26442
   -------------+---------------------------------
       Combined |     1016      516636      516636

   Unadjusted variance  4248348.00
   Adjustment for ties   -15999.58
                        ----------
   Adjusted variance    4232348.42

   H0: All_CFU(All_SA==0) = All_CFU(All_SA==1)
            z = -3.128
   Prob > |z| = 0.0018

   P{All_CFU(All_SA==0) > All_CFU(All_SA==1)} = 0.372

24 . display %4.3f 1-r(porder)
   0.628
```

```
25 . capture noisily ranksum All_CFU, by(All_SA) exact // shows why using spearman command
   number of observations must be <= 1,000 for exact option

26 .
27 . tabulate  bAll_CFU_100 All_SA

        1 =
    All_CFU     S. aurues all
   >=100; 0   reservoir samples
  otherwise         0          1       Total
-----------+----------------------+----------
          0       159          0         159
          1       805         52         857
-----------+----------------------+----------
      Total       964         52       1,016

28 . summarize  All_CFU  if All_SA == 1

    Variable |        Obs        Mean    Std. dev.       Min        Max
-------------+---------------------------------------------------------
     All_CFU |         52    1.91e+08    2.73e+08        500   1.62e+09

29 .
30 . roctab All_SA All_CFU, summary // added as recommended by Reviewer #2 comment #5

                      ROC                    Asymptotic normal
           Obs       area     Std. err.      [95% conf. interval]
     ------------------------------------------------------------
         1,016     0.6284       0.0365        0.55684     0.69988
```

```
31 . display "r(ub) - invnormal*r(se) = " r(ub) - ( invnormal(1-0.05/2)*r(se) + r(area) ) // asymptotic SE not extra info
   r(ub) - invnormal*r(se) = 0

32 .
33 . * Editor requested figure in R2 review
34 . roctab All_SA All_CFU, graph ///
   >     ytitle("Sensitivity of {it:S. aureus} Detection from Higher CFU")                  ///
   >     xtitle("False Positive Rate in Detection of {it:S. aureus} from Higher CFU")       ///
   >     note("") msize(small) mcolor("blue%30") mlcolor("blue")                            ///
   >     text(0.95 0.05 "Area under ROC curve: 0.628" "(95% CI: 0.557 to 0.700), N = 1016", ///
   >                    placement(se) justification(left) margin(small))

35 . graph export "Figure 1 v20.jpg", as(jpg) name("Graph") replace width(3600) height(2160) quality(100)
   file Figure 1 v20.jpg written in JPEG format

36 .
37 .
38 .
39 . * Second paragraph of the Results
40 . summarize                  Total_Saureus

       Variable |        Obs        Mean    Std. dev.       Min        Max
   -------------+---------------------------------------------------------
   Total_Saur~s |         81     .654321    1.097275          0          5

41 . display "Total among all samples: " r(mean)*r(N)
   Total among all samples: 53

42 .
43 . ratio (Total_CFU/Total_Saureus), vce(jackknife, nodots)

   Ratio estimation                            Number of obs = 81
                                               Replications  = 81

        _ratio_1: Total_CFU/Total_Saureus
```

```
    ───────────────────────────────────────────────────────────────
                 │               Jackknife
                 │      Ratio   std. err.     [95% conf. interval]
    ─────────────┼─────────────────────────────────────────────────
        _ratio_1 │   2.50e+09   5.33e+08      1.44e+09    3.56e+09
    ───────────────────────────────────────────────────────────────

44 . matrix list r(table) // extra digits and confidence interval

   r(table)[9,1]
             _ratio_1
         b  2.503e+09
        se  5.334e+08
         t  4.6922858
    pvalue  .00001099
        ll  1.441e+09
        ul  3.564e+09
        df         80
      crit  1.9900634
     eform          0

45 . display %4.2f r(table)[1,1] / 1E9
   2.50

46 . display %4.3f r(table)[2,1] / 1E9
   0.533

47 .
48 . * Reviewer #2 comment #3 inquires of inhierarchical structure. Adding to show effect (not in the results)
49 . ratio (Total_CFU/Total_Saureus)

   Ratio estimation                            Number of obs = 81

        _ratio_1: Total_CFU/Total_Saureus

    ───────────────────────────────────────────────────────────────
                 │              Linearized
                 │      Ratio   std. err.     [95% conf. interval]
    ─────────────┼─────────────────────────────────────────────────
        _ratio_1 │   2.50e+09   5.17e+08      1.47e+09    3.53e+09
    ───────────────────────────────────────────────────────────────
```

```
50 . display %4.2f r(table)[1,1] / 1E9
   2.50

51 . display %4.3f r(table)[2,1] / 1E9
   0.517

52 .
53 .
54 .
55 . * Third paragraph of the Results
56 . use "Temp_transmission.dta", clear

57 . describe, fullnames

   Contains data from Temp_transmission.dta
    Observations:            81
       Variables:            21                  18 Mar 2026 12:30
   ---------------------------------------------------------------------------------------------------------------------------
   Variable      Storage   Display    Value
       name         type    format    label      Variable label
   ---------------------------------------------------------------------------------------------------------------------------
   BoxNumber       str6    %9s                   Box number; N = 81 patient records
   mssa_transm     byte    %10.0g                MSSA transmission during case, 1 Yes 0 No
   mrsa_transm     byte    %10.0g                MRSA transmission during case, 1 Yes 0 No
   total_transm    byte    %8.0g                 Total S. aureus transmission events
   Sa_Start        byte    %10.0g                S. aureus isolates at start
   Sa_End          byte    %10.0g                S. aureus isolates at end
   vdo1_cfu        long    %10.0g                CFU: Valve dial at start
   aho1_cfu        long    %10.0g                CFU: Anesthesiologist hands at start
   aao1_cfu        long    %10.0g                CFU: Anesthesia resident hands at start
   pn1_cfu         long    %10.0g                CFU: Patient nares at start
   pa1_cfu         long    %10.0g                CFU: Patient axilla at start
   pg1_cfu         long    %10.0g                CFU Patient groin at start
   vdoe_cfu        long    %10.0g                CFU: Valve dial at end
   ahoe_cfu        long    %10.0g                CFU: Anesthesiologist hands at end
   aaoe_cfu        long    %10.0g                CFU: Anesthesia resident hands at end
   pne_cfu         long    %10.0g                CFU: Patient nares at end
   pae_cfu         long    %10.0g                CFU: Patient axilla at end
   pge_cfu         long    %10.0g                CFU: Patient groin at end
   le_cfu          long    %10.0g                CFU: Intravenous lumen at end
   premouseomnicell_cfu
                   long    %10.0g                CFU: Omnicell and mouse at start
```

```
postmouseomnicell_cfu
                long    %10.0g                 CFU: Omnicell and mouse at end
-------------------------------------------------------------------------------------
Sorted by:

.
. generate b_any_transm = (total_transm > 0) if total_transm != .

. generate b_Sa_Start   = (Sa_Start > 0) if Sa_Start != .

. generate b_Sa_End     = (Sa_End   > 0) if Sa_End   != .

.
. tabulate b_any_transm b_Sa_Start, row

+----------------+
| Key            |
|----------------|
|   frequency    |
| row percentage |
+----------------+

b_any_tran |      b_Sa_Start
        sm |         0          1 |     Total
-----------+----------------------+----------
         0 |        53         10 |        63
           |     84.13      15.87 |    100.00
-----------+----------------------+----------
         1 |        10          8 |        18
           |     55.56      44.44 |    100.00
-----------+----------------------+----------
     Total |        63         18 |        81
           |     77.78      22.22 |    100.00
```

```
64 . tabulate b_any_transm b_Sa_End, column
```

Key
frequency
column percentage

b_any_transm	b_Sa_End 0	1	Total
0	**57**<br>**98.28**	**6**<br>**26.09**	**63**<br>**77.78**
1	**1**<br>**1.72**	**17**<br>**73.91**	**18**<br>**22.22**
Total	**58**<br>**100.00**	**23**<br>**100.00**	**81**<br>**100.00**

```
65 .
66 . generate b_Sa_Either = 0

67 . replace  b_Sa_Either = . if Sa_Start == . & Sa_End == .
   (0 real changes made)

68 . replace  b_Sa_Either = 1 if Sa_Start >= 1 | Sa_End >= 1
   (28 real changes made)
```

```
69 . tabulate b_any_transm b_Sa_Either, column
```

Key
frequency
column percentage

b_any_transm	b_Sa_Either 0	1	Total
0	**53**	**10**	**63**
	**100.00**	**35.71**	**77.78**
1	**0**	**18**	**18**
	**0.00**	**64.29**	**22.22**
Total	**53**	**28**	**81**
	**100.00**	**100.00**	**100.00**

```
70 .
71 .
72 .
73 . * Fourth paragraph of the Results
74 . * One-sided tests because would not deliberately contaminate to prevent S. aureus transmission
75 . generate byte ms_msra_transm_any = 0

76 . replace       ms_msra_transm_any = 1 if mssa_transm == 1 | mrsa_transm == 1
   (18 real changes made)
```

```
77 .
78 . ranksum postmouseomnicell_cfu, by(ms_msra_transm_any) porder

   Two-sample Wilcoxon rank-sum (Mann–Whitney) test

   ms_msra_tr~y |       Obs    Rank sum    Expected
   -------------+---------------------------------
              0 |        58      2030.5        2146
              1 |        15       670.5         555
   -------------+---------------------------------
       Combined |        73        2701        2701

   Unadjusted variance     5365.00
   Adjustment for ties       -0.58
                          ----------
   Adjusted variance       5364.42

   H0: postmo~u(ms_msr~y==0) = postmo~u(ms_msr~y==1)
            z = -1.577
   Prob > |z| = 0.1148
   Exact prob = 0.1162

   P{postmo~u(ms_msr~y==0) > postmo~u(ms_msr~y==1)} = 0.367

79 . display "unadjusted "             r(p_exact)/2
   unadjusted .05809564

80 . display "adjusted " min(0.999, 15*r(p_exact)/2)
   adjusted .87143463
```

```
81 . ranksum ahoe_cfu, by(ms_msra_transm_any) porder

   Two-sample Wilcoxon rank-sum (Mann–Whitney) test

   ms_msra_tr~y |        Obs    Rank sum    Expected
   -------------+---------------------------------
              0 |         59      2140.5        2242
              1 |         16       709.5         608
   -------------+---------------------------------
       Combined |         75        2850        2850

   Unadjusted variance     5978.67
   Adjustment for ties      -84.70
                        ----------
   Adjusted variance       5893.96

   H0: ahoe_cfu(ms_msr~y==0) = ahoe_cfu(ms_msr~y==1)
            z = -1.322
   Prob > |z| = 0.1861
   Exact prob = 0.1895

   P{ahoe_cfu(ms_msr~y==0) > ahoe_cfu(ms_msr~y==1)} = 0.392

82 . display "adjusted " min(0.999, 14*r(p_exact)/2) // Holm-Bonferroni sequential
   adjusted .999

83 . ranksum aao1_cfu, by(ms_msra_transm_any) porder

   Two-sample Wilcoxon rank-sum (Mann–Whitney) test

   ms_msra_tr~y |        Obs    Rank sum    Expected
   -------------+---------------------------------
              0 |         62        2494        2511
              1 |         18         746         729
   -------------+---------------------------------
       Combined |         80        3240        3240

   Unadjusted variance     7533.00
   Adjustment for ties       -6.53
                        ----------
   Adjusted variance       7526.47
```

```
   H0: aao1_cfu(ms_msr~y==0) = aao1_cfu(ms_msr~y==1)
            z = -0.196
   Prob > |z| = 0.8446
   Exact prob = 0.8483

   P{aao1_cfu(ms_msr~y==0) > aao1_cfu(ms_msr~y==1)} = 0.485

84 . display "adjusted " min(0.999, 13*r(p_exact)/2)
   adjusted .999

85 . ranksum aho1_cfu, by(ms_msra_transm_any) porder

   Two-sample Wilcoxon rank-sum (Mann-Whitney) test

   ms_msra_tr~y |      Obs    Rank sum    Expected
   -------------+---------------------------------
              0 |       60        2270        2280
              1 |       15         580         570
   -------------+---------------------------------
       Combined |       75        2850        2850

   Unadjusted variance     5700.00
   Adjustment for ties      -56.19
                          ----------
   Adjusted variance       5643.81

   H0: aho1_cfu(ms_msr~y==0) = aho1_cfu(ms_msr~y==1)
            z = -0.133
   Prob > |z| = 0.8941
   Exact prob = 0.8969

   P{aho1_cfu(ms_msr~y==0) > aho1_cfu(ms_msr~y==1)} = 0.489
```

```
86 . display "adjusted " min(0.999, 12*r(p_exact)/2)
   adjusted .999

87 . ranksum pge_cfu , by(ms_msra_transm_any) porder

   Two-sample Wilcoxon rank-sum (Mann–Whitney) test

   ms_msra_tr~y |      Obs    Rank sum    Expected
   -------------+---------------------------------
              0 |       40        1101        1080
              1 |       13         330         351
   -------------+---------------------------------
       Combined |       53        1431        1431

   Unadjusted variance     2340.00
   Adjustment for ties     -424.34
                         ----------
   Adjusted variance       1915.66

   H0: pge_cfu(ms_msr~y==0) = pge_cfu(ms_msr~y==1)
            z =  0.480
   Prob > |z| = 0.6314
   Exact prob = 0.6474

   P{pge_cfu(ms_msr~y==0) > pge_cfu(ms_msr~y==1)} = 0.540

88 . display 1 - (r(p_exact) / 2)                    // one-sided and reversing
   .67628597

89 . ranksum premouseomnicell_cfu , by(ms_msra_transm_any) porder

   Two-sample Wilcoxon rank-sum (Mann–Whitney) test

   ms_msra_tr~y |      Obs    Rank sum    Expected
   -------------+---------------------------------
              0 |       60      2315.5        2280
              1 |       15       534.5         570
   -------------+---------------------------------
       Combined |       75        2850        2850
```

```
   Unadjusted variance     5700.00
   Adjustment for ties       -0.65
                          ----------
   Adjusted variance       5699.35

   H0: premou~u(ms_msr~y==0) = premou~u(ms_msr~y==1)
            z =  0.470
   Prob > |z| = 0.6382
   Exact prob = 0.6439

   P{premou~u(ms_msr~y==0) > premou~u(ms_msr~y==1)} = 0.539

90 . display 1 - (r(p_exact) / 2)
   .67807048

91 . ranksum pa1_cfu , by(ms_msra_transm_any) porder

   Two-sample Wilcoxon rank-sum (Mann-Whitney) test

   ms_msra_tr~y |      Obs    Rank sum    Expected
   -------------+---------------------------------
              0 |       40        1112        1080
              1 |       13         319         351
   -------------+---------------------------------
       Combined |       53        1431        1431

   Unadjusted variance     2340.00
   Adjustment for ties       -0.38
                          ----------
   Adjusted variance       2339.62

   H0: pa1_cfu(ms_msr~y==0) = pa1_cfu(ms_msr~y==1)
            z =  0.662
   Prob > |z| = 0.5082
   Exact prob = 0.5162

   P{pa1_cfu(ms_msr~y==0) > pa1_cfu(ms_msr~y==1)} = 0.562
```

```
92 . display 1 - (r(p_exact) / 2)
   .74192134

93 . ranksum pne_cfu , by(ms_msra_transm_any) porder

   Two-sample Wilcoxon rank-sum (Mann–Whitney) test

   ms_msra_tr~y |        Obs    Rank sum    Expected
   -------------+---------------------------------
              0 |         39        1071      1033.5
              1 |         13         307       344.5
   -------------+---------------------------------
       Combined |         52        1378        1378

   Unadjusted variance     2239.25
   Adjustment for ties       -0.38
                          ----------
   Adjusted variance       2238.87

   H0: pne_cfu(ms_msr~y==0) = pne_cfu(ms_msr~y==1)
            z =  0.793
   Prob > |z| = 0.4281
   Exact prob = 0.4389

   P{pne_cfu(ms_msr~y==0) > pne_cfu(ms_msr~y==1)} = 0.574

94 . display 1 - (r(p_exact) / 2)
   .78054821

95 . ranksum aaoe_cfu, by(ms_msra_transm_any) porder

   Two-sample Wilcoxon rank-sum (Mann–Whitney) test

   ms_msra_tr~y |        Obs    Rank sum    Expected
   -------------+---------------------------------
              0 |         61      2477.5      2409.5
              1 |         17       603.5       671.5
   -------------+---------------------------------
       Combined |         78        3081        3081
```

```
   Unadjusted variance     6826.92
   Adjustment for ties      -10.96
                          ----------
   Adjusted variance       6815.95

   H0: aaoe_cfu(ms_msr~y==0) = aaoe_cfu(ms_msr~y==1)
            z =  0.824
   Prob > |z| = 0.4101
   Exact prob = 0.4153

   P{aaoe_cfu(ms_msr~y==0) > aaoe_cfu(ms_msr~y==1)} = 0.566

96 . display 1 - (r(p_exact) / 2)
   .79234516

97 . ranksum pn1_cfu , by(ms_msra_transm_any) porder

   Two-sample Wilcoxon rank-sum (Mann-Whitney) test

   ms_msra_tr~y |      Obs    Rank sum    Expected
   -------------+---------------------------------
              0 |       40        1122        1080
              1 |       13         309         351
   -------------+---------------------------------
       Combined |       53        1431        1431

   Unadjusted variance     2340.00
   Adjustment for ties       -0.38
                          ----------
   Adjusted variance       2339.62

   H0: pn1_cfu(ms_msr~y==0) = pn1_cfu(ms_msr~y==1)
            z =  0.868
   Prob > |z| = 0.3852
   Exact prob = 0.3955

   P{pn1_cfu(ms_msr~y==0) > pn1_cfu(ms_msr~y==1)} = 0.581
```

```
 98 . display 1 - (r(p_exact) / 2)
    .80226758

 99 . ranksum le_cfu  , by(ms_msra_transm_any) porder

    Two-sample Wilcoxon rank-sum (Mann–Whitney) test

    ms_msra_tr~y |      Obs    Rank sum    Expected
    -------------+---------------------------------
               0 |       63      2642.5        2583
               1 |       18       678.5         738
    -------------+---------------------------------
        Combined |       81        3321        3321

    Unadjusted variance     7749.00
    Adjustment for ties    -3474.97
                          ----------
    Adjusted variance       4274.03

    H0: le_cfu(ms_msr~y==0) = le_cfu(ms_msr~y==1)
             z =  0.910
    Prob > |z| = 0.3628
    Exact prob = 0.3326

    P{le_cfu(ms_msr~y==0) > le_cfu(ms_msr~y==1)} = 0.552

100 . display 1 - (r(p_exact) / 2)
    .83369928

101 . ranksum vdo1_cfu, by(ms_msra_transm_any) porder

    Two-sample Wilcoxon rank-sum (Mann–Whitney) test

    ms_msra_tr~y |      Obs    Rank sum    Expected
    -------------+---------------------------------
               0 |       63      2695.5        2583
               1 |       18       625.5         738
    -------------+---------------------------------
        Combined |       81        3321        3321
```

```
Unadjusted variance     7749.00
Adjustment for ties      -15.75
                     ----------
Adjusted variance       7733.25

H0: vdo1_cfu(ms_msr~y==0) = vdo1_cfu(ms_msr~y==1)
         z =  1.279
Prob > |z| = 0.2008
Exact prob = 0.2036

P{vdo1_cfu(ms_msr~y==0) > vdo1_cfu(ms_msr~y==1)} = 0.599

102 . display 1 - (r(p_exact) / 2)
    .89818825

103 . ranksum vdoe_cfu, by(ms_msra_transm_any) porder

Two-sample Wilcoxon rank-sum (Mann-Whitney) test

ms_msra_tr~y |      Obs    Rank sum    Expected
-------------+---------------------------------
           0 |       63      2698.5        2583
           1 |       18       622.5         738
-------------+---------------------------------
    Combined |       81        3321        3321

Unadjusted variance     7749.00
Adjustment for ties       -7.88
                     ----------
Adjusted variance       7741.12

H0: vdoe_cfu(ms_msr~y==0) = vdoe_cfu(ms_msr~y==1)
         z =  1.313
Prob > |z| = 0.1893
Exact prob = 0.1919

P{vdoe_cfu(ms_msr~y==0) > vdoe_cfu(ms_msr~y==1)} = 0.602
```

```
104 . display 1 - (r(p_exact) / 2)
    .90403353

105 . ranksum pae_cfu , by(ms_msra_transm_any) porder

    Two-sample Wilcoxon rank-sum (Mann–Whitney) test

    ms_msra_tr~y |        Obs    Rank sum    Expected
    -------------+-----------------------------------
               0 |         40      1146.5        1080
               1 |         13       284.5         351
    -------------+-----------------------------------
        Combined |         53        1431        1431

    Unadjusted variance     2340.00
    Adjustment for ties       -0.85
                          ----------
    Adjusted variance       2339.15

    H0: pae_cfu(ms_msr~y==0) = pae_cfu(ms_msr~y==1)
             z =  1.375
    Prob > |z| = 0.1691
    Exact prob = 0.1728

    P{pae_cfu(ms_msr~y==0) > pae_cfu(ms_msr~y==1)} = 0.628

106 . display 1 - (r(p_exact) / 2)
    .91358174

107 . ranksum pg1_cfu , by(ms_msra_transm_any) porder

    Two-sample Wilcoxon rank-sum (Mann–Whitney) test

    ms_msra_tr~y |        Obs    Rank sum    Expected
    -------------+-----------------------------------
               0 |         40      1200.5        1080
               1 |         13       230.5         351
    -------------+-----------------------------------
        Combined |         53        1431        1431
```

```
    Unadjusted variance     2340.00
    Adjustment for ties       -2.74
                          ----------
    Adjusted variance       2337.26

    H0: pg1_cfu(ms_msr~y==0) = pg1_cfu(ms_msr~y==1)
             z =  2.492
    Prob > |z| = 0.0127
    Exact prob = 0.0116

    P{pg1_cfu(ms_msr~y==0) > pg1_cfu(ms_msr~y==1)} = 0.732

108 . display "two-sided " r(p_exact)
    two-sided .0116017

109 . display 1 - (r(p_exact) / 2)
    .99419915

110 .
111 .
112 . * Added for Reviewer #2 comment #3 requesting effect sizes in results
113 . capture program drop mean_sd_auc

114 . quietly {

115 .
116 . ds *_cfu                      // accumulates the 15 CFU variables (i.e., reservoirs)
    vdo1_cfu      aao1_cfu      pa1_cfu       vdoe_cfu      aaoe_cfu      pae_cfu       le_cfu        postmouseo~u
    aho1_cfu      pn1_cfu       pg1_cfu       ahoe_cfu      pne_cfu       pge_cfu       premouseom~u
```

```
117 . global cfu_vars `r(varlist)'  // what used in the preceding program

118 . mean_sd_auc                    // running the program once for (new) second to last sentence

119 . display "Mean of the 15 AUCs: " %6.3f _b[mean_auc] " SD: "%6.3f e(sd_auc) " N cases as check: " e(N)
    Mean of the 15 AUCs:  0.453 SD:  0.090 N cases as check: 81

120 . jackknife _b[mean_auc] : mean_sd_auc // new final sentence of the last paragraph of the results
    (running mean_sd_auc on estimation sample)

    Jackknife replications (81): .........10.........20.........30.........40.........50.........60.........70.........80. done

    Jackknife results                                             Number of obs = 81
                                                                  Replications  = 81


          Command: mean_sd_auc
            _jk_1: _b[mean_auc]
              n(): e(N)

    -------------------------------------------------------------------------------
                 |              Jackknife
                 |  Coefficient  std. err.      t    P>|t|     [95% conf. interval]
    -------------+-----------------------------------------------------------------
           _jk_1 |    .4527871   .0361212    12.54   0.000     .3809037    .5246705
    -------------------------------------------------------------------------------

121 . test _jk_1 = 0.5

     ( 1)  _jk_1 = .5

           F(  1,    80) =    1.71
                Prob > F =    0.1949
```
